# A compact and efficient AES-32GF for encryption in small IoT devices

**DOI:** 10.1016/j.mex.2023.102491

**Published:** 2023-11-21

**Authors:** Sumit Singh Dhanda, Poonam Jindal, Brahmjit Singh, Deepak Panwar

**Affiliations:** a Department of Electronics and Communication Engineering, National Institute of Technology Kurukshetra, India; b Manipal University Jaipur, Jaipur, India

**Keywords:** Advance encryption scheme (AES), Internet of things (IoT), Field programmable gate arrays (FPGA), 32-bit data path, Information security, Lightweight AES-32GF

## Abstract

The phenomenal growth of resource constrained devices in IoT set ups has motivated the researchers to develop solutions for securing information flow. In this paper, we present a compact and efficient field programmable gate array (FPGA) implementation of AES with 32-bit data-path named, AES-32GF. The implementation is carried out on different Xilinx FPGAs. In FPGAs, utilization of slices and look up tables (LUTs) reflect on the compactness of the design. Numerical results show that lesser resources are required with smaller data path in comparison with the original standard. With the help of data path compression and Galois field implementation of the s-box resource consumption is minimized. S-box is the most resource consuming component in the AES structure. In our implementation, Artix-7 series FPGA for the same. It results in significant resource savings. In comparison to unrolled AES-128 architecture, it achieves 87 % resource savings. With 595 slices and 2.004 Gbps throughput, AES-32GF cipher achieves an efficiency of 3.37 Mbps/slice. It outperforms other designs in terms of efficiency.

• A compact and efficient FPGA implementation of AES with 32-bit data-path has been proposed.
• The proposed design utilizes data path compression and Galois field implementation of the s-box to minimize resource consumption.
• With 595 slices and 2.004 Gbps throughput, AES-32GF cipher achieves an efficiency of 3.37 Mbps/slice.

## Graphical abstract

**Figure:**
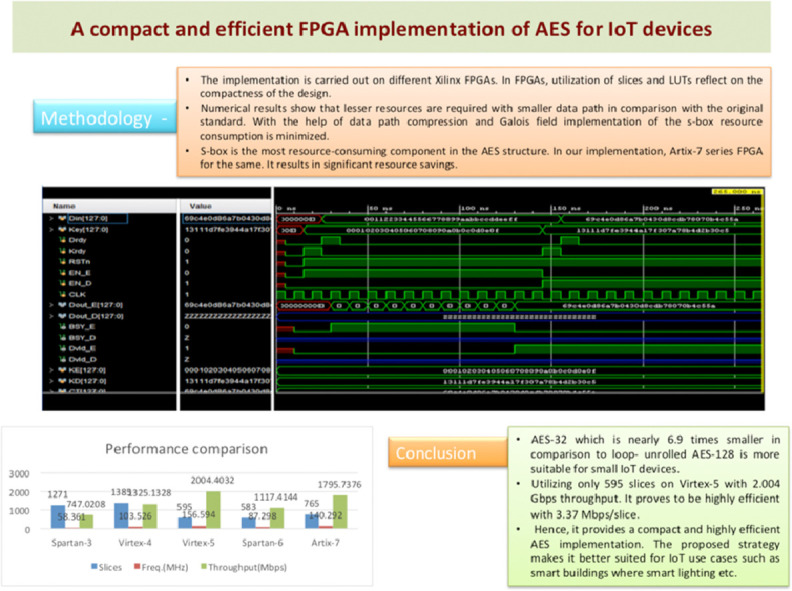

Specifications table

**Table:** 

Subject area:	Computer Science
More specific subject area:	Lightweight Cryptography
Name of your method:	Lightweight AES-32GF
Name and reference of original method:	AES-128/ FIPS-196 list the full bibliographic details of any key reference(s) that describe the original method has been customized
Resource availability:	Software: https://www.xilinx.com/support/download.html Hardware: https://www.digikey.in/en/products/detail/xilinx-inc/XC5VLX50T-2FF1136I/1,768,595 AES-128: https://nvlpubs.nist.gov/nistpubs/fips/nist.fips.197.pdf

## Method details

### AES-32GF for resource-constrained devices

AES adopted as a standard for the security under FIPS-197 by NIST in 2001 [1]. Advanced Encryption Standard (AES) is a widely used block cipher offering significant benefits in providing security [[2], [3]]. Cipher is suited for both hardware and software platforms but hardware implementation is preferred for high data rates. Field programmable gate arrays (FPGA) or application specific integrated chips (ASIC) are two options available for the hardware implementation. But there is a major issue regarding this design. It utilizes a lot of resources for its hardware implementation. This implementation results in a high area design that is not suited for the small IoT devices which have limited resources in terms of memory, computation and power etc.

**Fig. 1:**
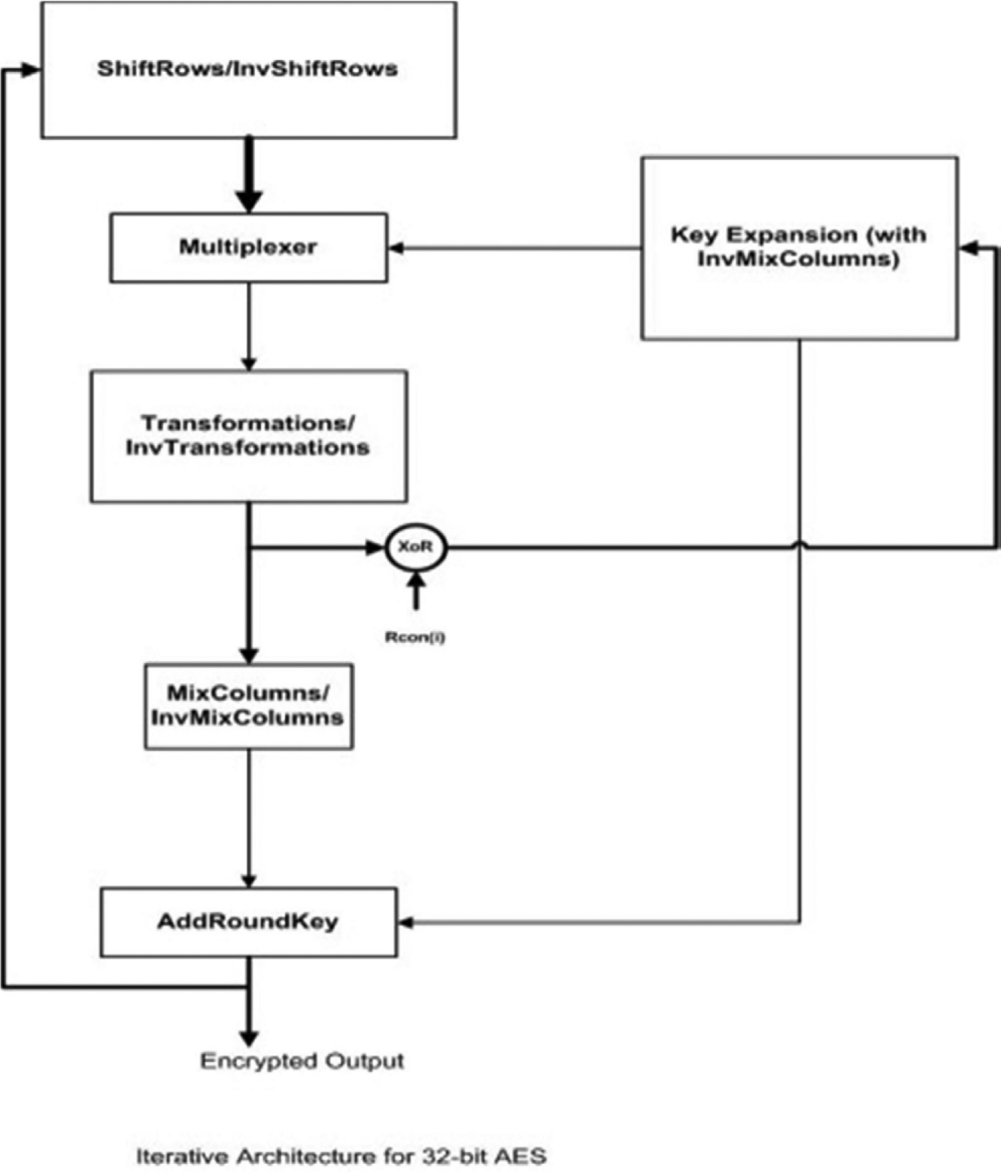
Iterative architecture AES-32GF.

The paper uses a three-step approach, as shown in Fig. 1, to adapt standard AES design for the resource-constrained devices.

• In first step, the data path of the standard AES design has been compressed to 32-bit width. It uses an iterative architecture of AES that completes one AES-round in one clock cycle. Hence, to process a 128-bit block it will take 10 cycles.
• In second step, the S-box has been implemented in galois field. Using simple transformation ax+*B*. It minimizes the resources required to implement SubBytes round.
• In third step, redundancy of MixColumns multiplication matrix is utilized.
• In fourth step, a separate S-box is provided for the key schedule to minimize the latency of the design.

Two important operations are on-the-fly generation of keys and use of separate S-box for the key generation which has helped in the improving throughput. 128-bit input supplied AES-32GF module is divided into four separate blocks of 32-bits each. SubBytes operation is applied on this 32-bit block. ShiftRows operation provides confusion with byte shuffling operations. It is the only non-linear operation present in the AES structure. It changes the sequence of the bytes. After wards, MixColumns operation is performed which are 32-bit wide and four in number. It utilizes the redundancy present in the matrix coefficient. Final operation is Add round key and a separate SubBytes unit is used for the same. Here, the calculation is done on the fly. The SubBytes operation utilizes either galois field (GF) arithmetic or the S-Box can be stored as look up table (LUT). Here, S-box is implemented with galois field arithmetic. In this architecture, the encryption and decryption utilize the same data-path and resources. This has been enabled by the use of multiplexers. Key Expansion unit also utilizes the separate S-box which reduces latency in throughput.

**Fig. 2:**
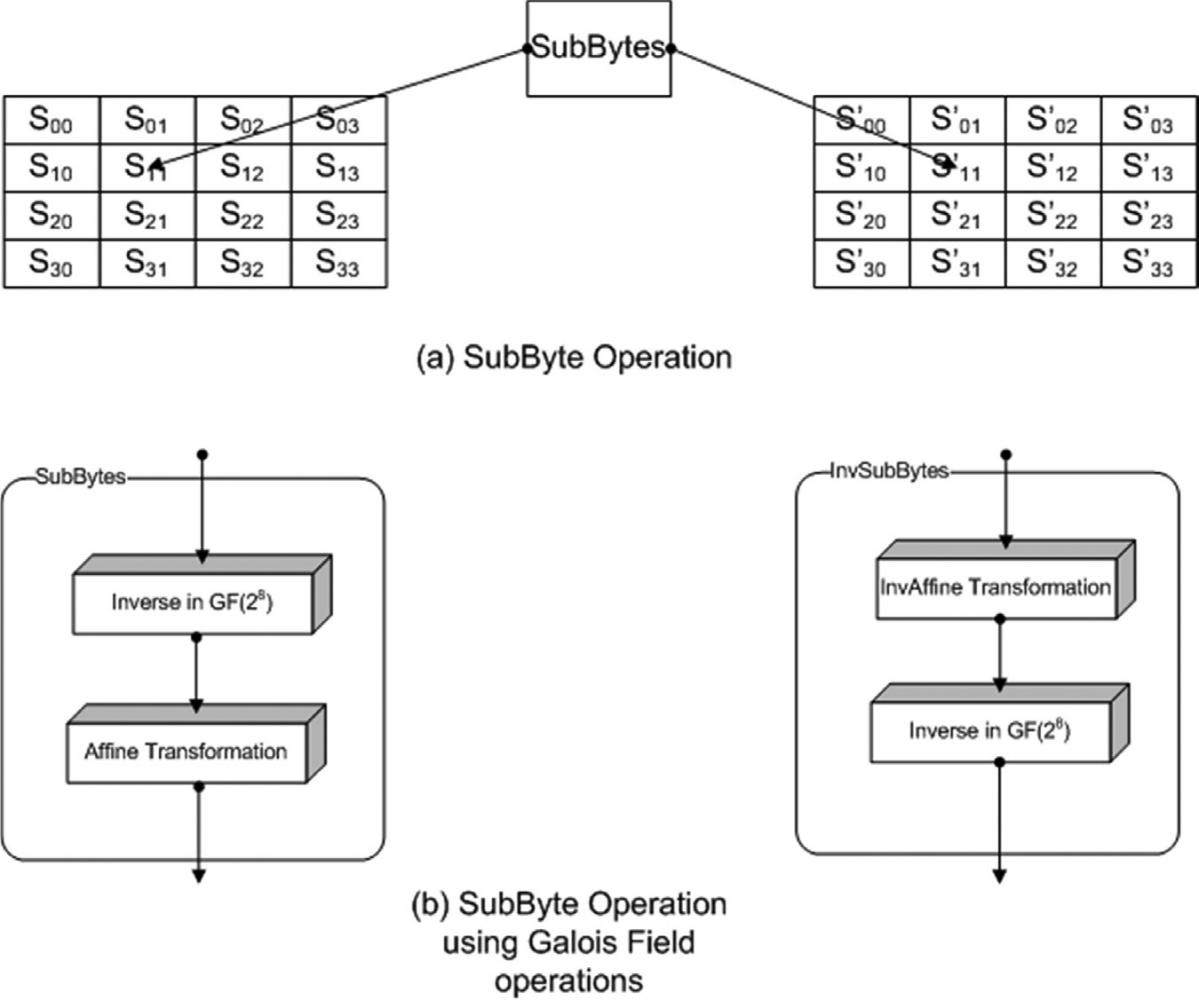
SubBytes calculations in GF(2^8^).

**Fig. 3:**
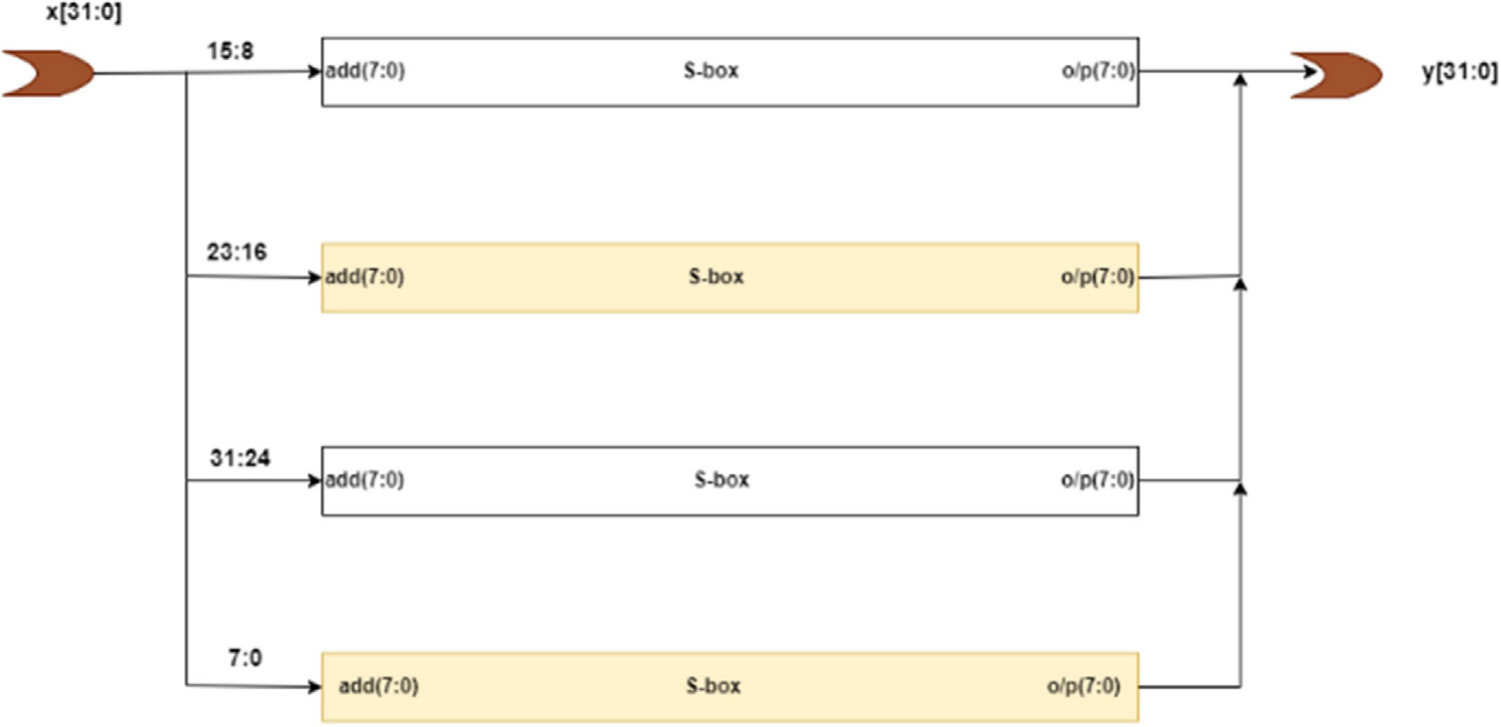
SubBytes for AES-32GF.

There are two methods for the implementation of SubBytes in AES. One is to use look up table to store these substitution bytes. The main drawback of this method is that it requires 256 bytes for the storage of each copy of table. All the 16 bytes of a block can be processed in parallel which will require 16 copies of this table. It results in high resource consumption. Second, is to calculate SubBytes in galois field. This second method is employed for the minimization of resources. Here, the inverse of the byte is calculated in the GF(2^8^) field and multiplied with a matrix (affine transformation) to obtain the substitution byte. It is shown in the Fig. 2. This method is improved by using the D. Canright's S-box for the SubByte calculation. It breaks the calculations in to the smaller fields and calculates these into GF(2^4^) and further into GF(2^2^)i.e. GF(((2^2^)^2^)^2^). In this way, the size of the SubBytes becomes smaller. Four 8-bit S-box are used for the SubBytes calculations. The structure is shown in the Fig. 3.

**Fig. 4:**
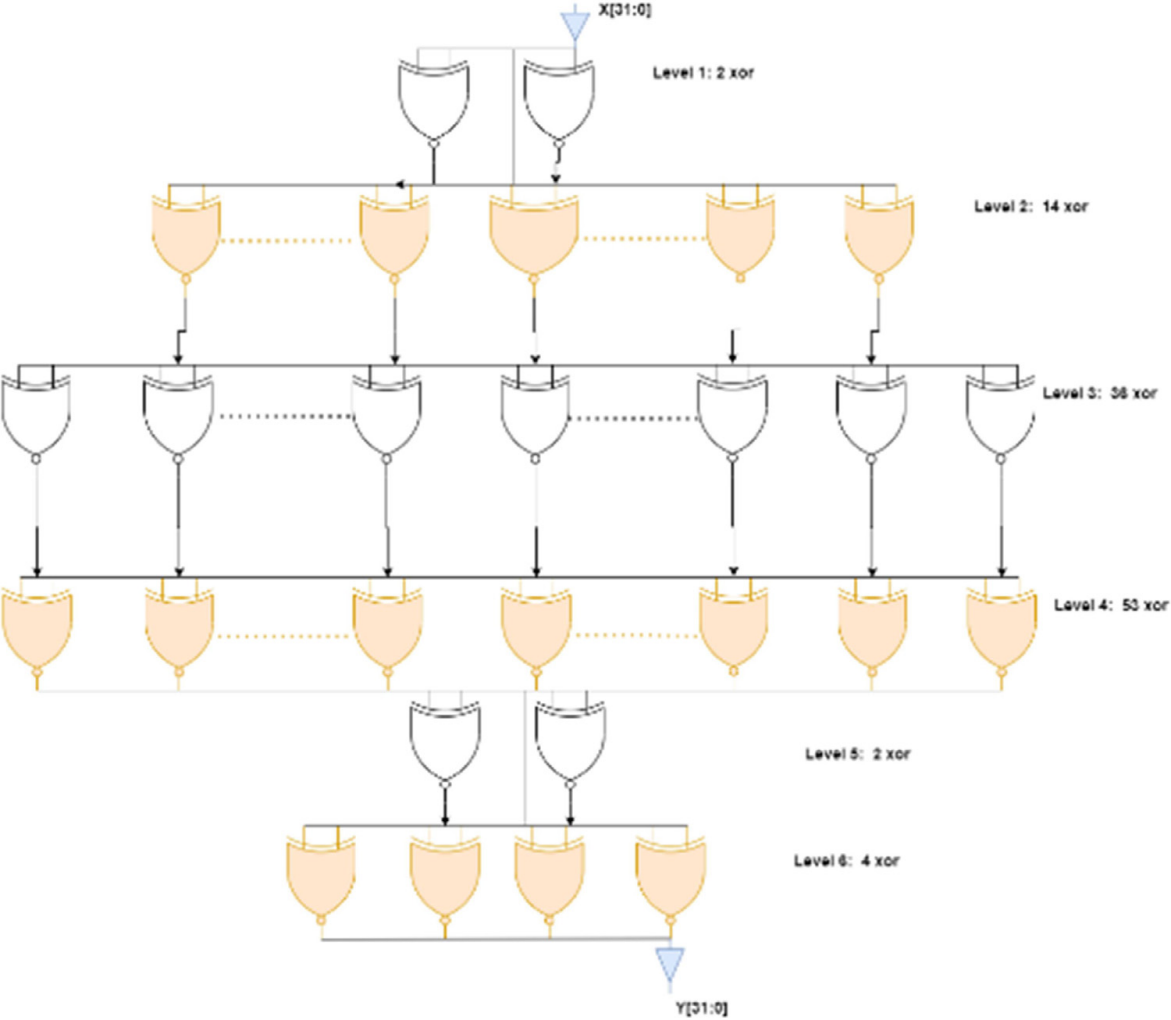
MixColumns for AES-32GF.

Second method used for this constrained implementation is to exploit the redundancy of MixColumns operation. It can be seen in the Fig. 4 where the redundancy of the MixColumn matrix is utilized. As the same coefficients are used in each row of MixColumn matrix. These coefficients can be reused for the same. The resultant MixColumn is shown in the Fig. 4 which has 6 levels of logic for its implementations.

Third Method used for the reduction in latency is the use of separate S-box for the key schedule. It helps in on-the-fly calculation of the round keys, removes the need of the storage and cut downs the resource requirement.

## Area savings achieved with AES-32 GF

The resource consumption of the implemented design is reported in Table 1. The area savings are presented in Table 2, which depictss the resource consumption of three designs. First is the AES-128, i.e. loop unrolled architecture for AES. It uses 128-bit data-path. Second is AES-32, the proposed design i.e. AES with 32 bit data path. It has an iterative architecture but SubBytes calculations are done in GF(2^8^). Finally, AES with 32-bit input but 128-bit data path has also been implemented. It uses all the methods mentioned in the previous section. All have been implemented on the same Artix-7 FPGA using Xilinx PlanAhead software. Table 2 shows that AES-128 consumes 2668 slices and a total of 9571 LUTs. Hence, a total improvement of 86.47 percent is achieved by AES-32. Similarly, a comparison with AES-32 shows an improvement of 14.85 percent. This implementation consumes 424 slices and 1231 LUTs. Results of Table 1 are depicted in Fig. 6 which clearly emphasize the reduction achieved by the iterative design with compressed data path.

**Table 1:** Resource utilization of AES-32 on Artix-7 FPGA.

Module	Slices	LUTs	Slice Registers	F/F
AES-32 bit	361	1033	395	110
Data-path	292	912		–
Key Expansion (with S-box)	52	100	–	
Out of total: Slice-M	151			
Slice-L	210

**Table 2:** Comparison of three designs amongst each other.

Design	Slices	LUTs	Area Improvement by 32-bit AES GF w.r.t to design (%)
AES-128 (loop unrolled architecture)	2669	9571	86.47
AES-32-bit	424	1231	14.85
AES-32GF	361	1033	–

## Resource consumption analysis of AES-32 GF and its comparison with the existing designs

**Fig. 5:**
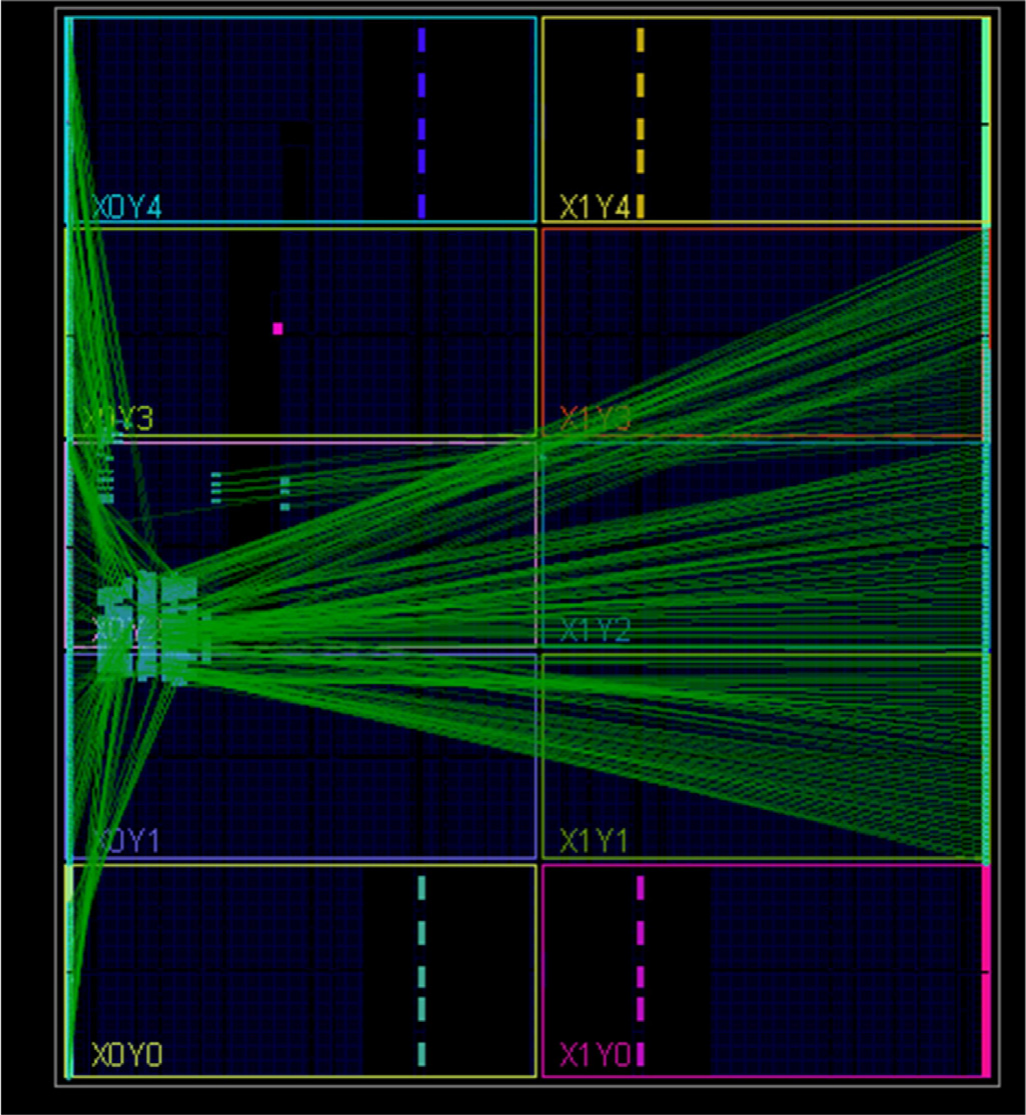
AES with 32-bit data path on Artix-7 FPGA.

**Fig. 6:**
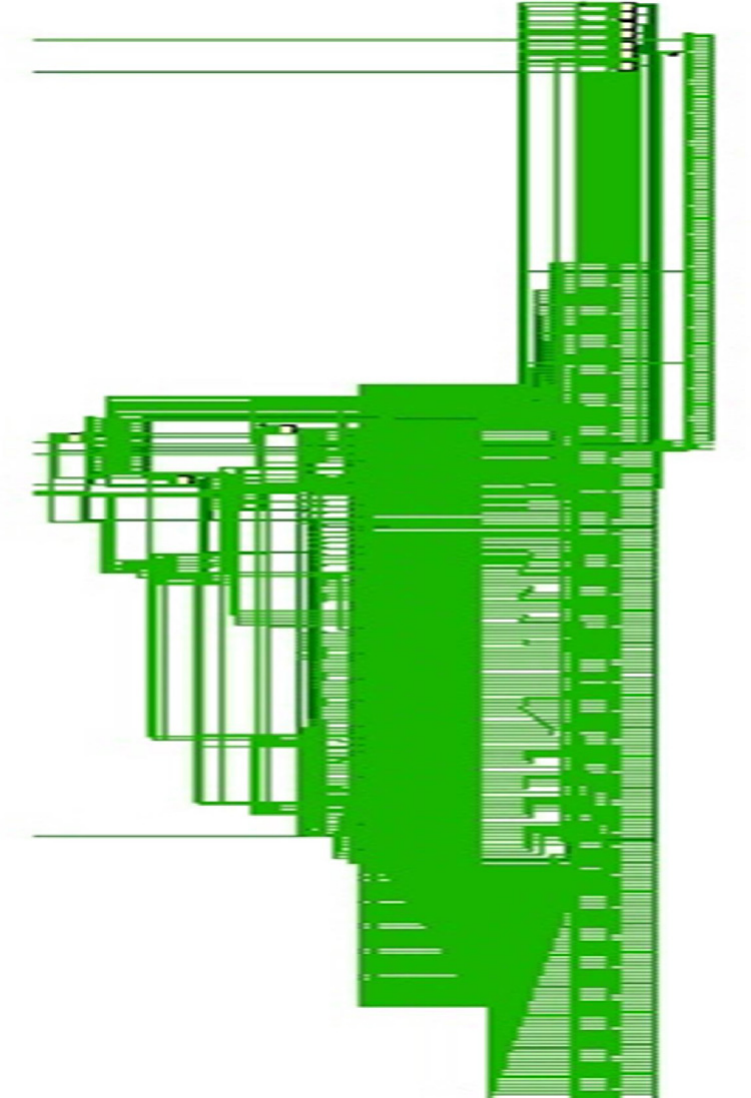
Synthesized schematic on Artix-7 FPGA.

The designs are synthesized in Xilinx Vivado software version 2014 and implementation is carried out on Artix-7 FPGA. While the implementation is carried out in PlanAhead software for the Spartan-3, Spartan-6 and Virtex-4, Virtex-5 and Artix-7 FPGAs for comparison purposes. Fig. 5 depicts the implemented design on Artix-7 FPGA using Vivado software while Fig. 6 represent the top-level schematic of the AES-32 on the same. AES-128 has been implemented as loop unrolled architecture while as mentioned earlier; AES-32 is implemented as the iterative architecture.

**Fig. 7:**
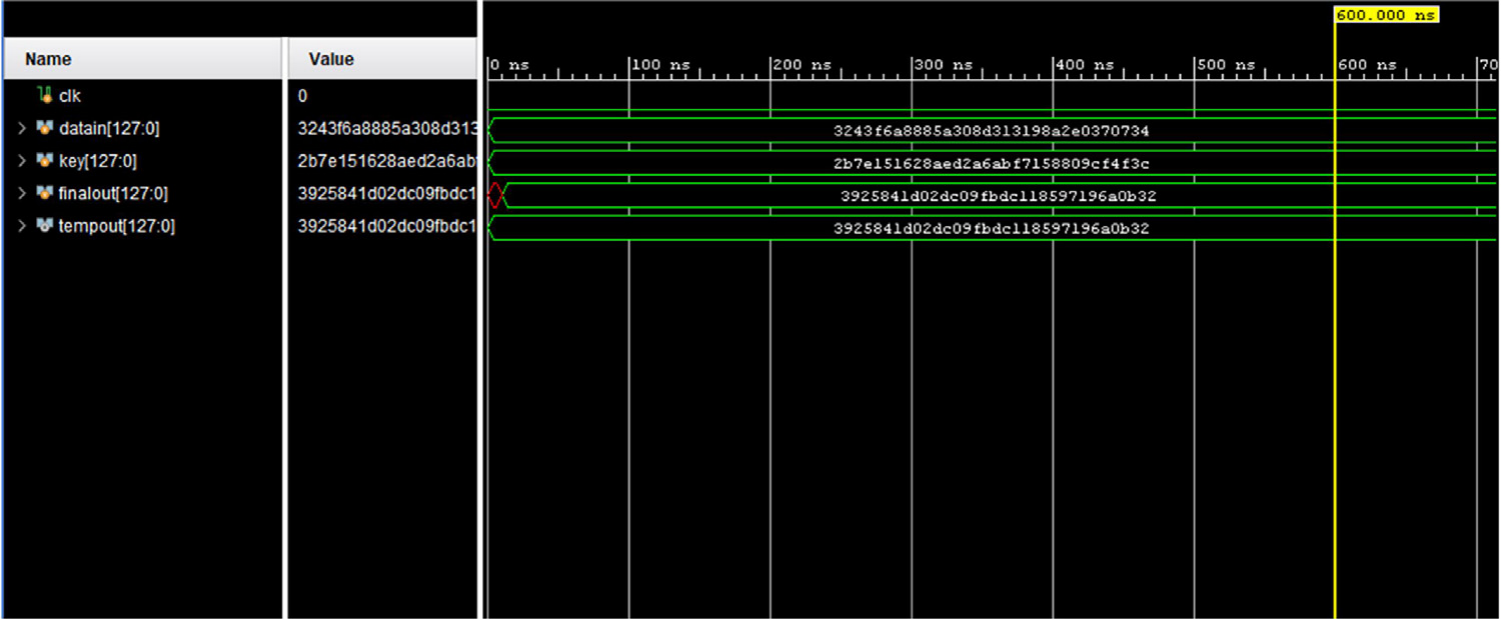
Simulation waveform for AES-128.

**Fig. 8:**
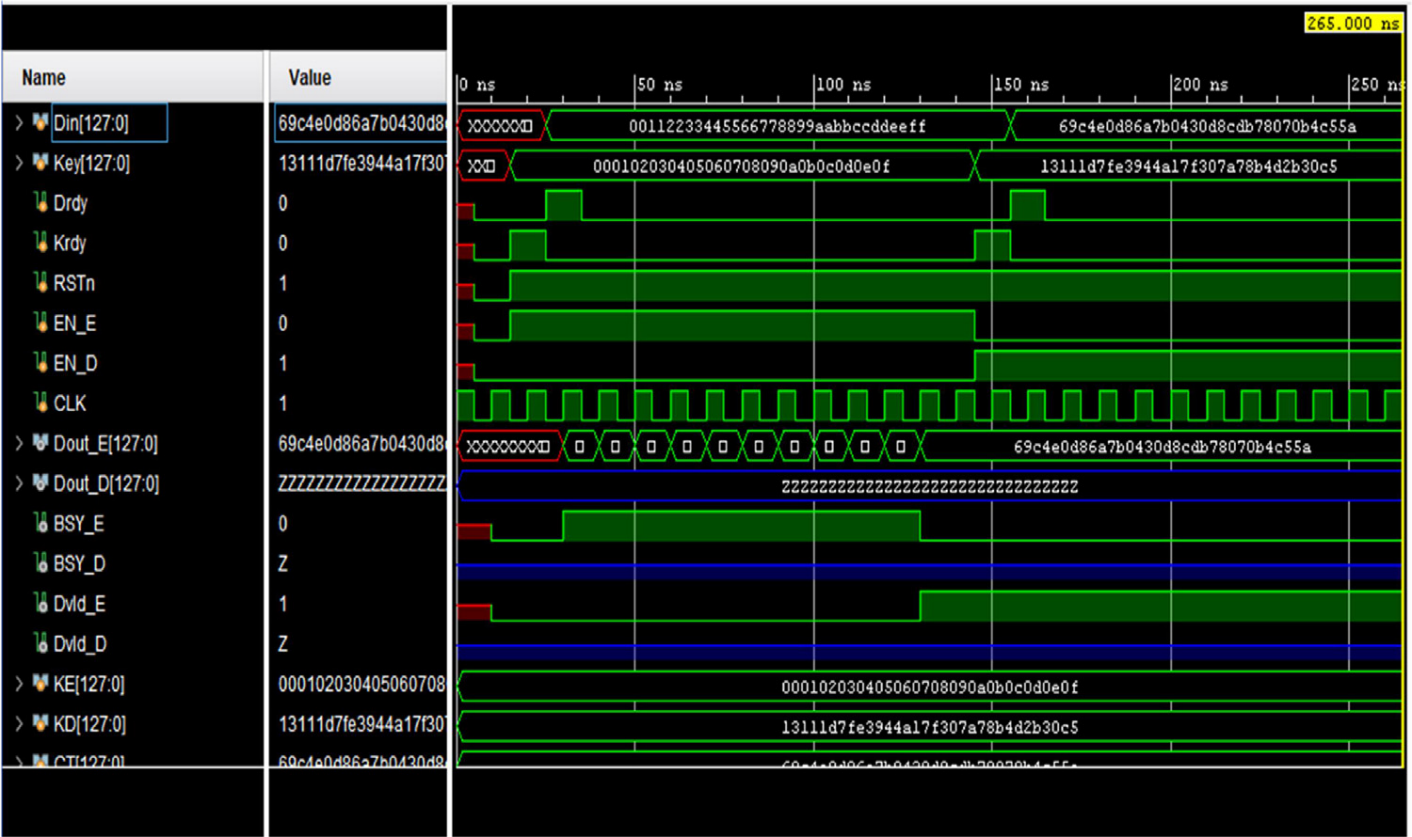
Simulation waveform for AES-32.

Fig. 7 depicts the simulation waveform of the loop unrolled architecture for AES. It confirms that the design is working properly and results are correct. Fig. 8 presents the simulation waveform for the AES-32. Various parameters and signals used in design are also visible in this.

Both the designs complete the calculations in 10 cycles. Hence, the designs are able to provide high throughput. But the loop unrolled architecture is able to achieve higher maximum frequency of operation as compared to iterative architecture. AES-32 consumes 1033 LUTs while AES-128 consumes 9571 LUTs hence, there is a large difference between these two designs in terms of resource consumption.

Different applications in Internet of Things (IoT) call for different security solution. One type of solution can not fit in all the situations. As in case of vehicular or Unmanned Arieal Vehicles (UAV) control latency is critical parameter in design of cipher. Hence, a cipher must be designed for high throughput with low cycle count. But in case of smart building application the main concern is securing the small sensors and their information from hackers. Here main parameter is to adapt cipher to these small devices. Hence, minimizing the resource consumption and area requirement should be the most important concern. The low area consumption (slice consumption) makes AES-32 suitable for the implementation in the sensors which can be used for the information security in smart buildings in smart lighting, AC control etc. But increased latency unsuitable for the applications such as real time vehicular control or smart-grid applications.

For a comparison of the area savings in AES-32, we have implemented AES-128 as loop unrolled architecture. AES-32 is implemented as the iterative architecture.

A division of resource consumption by AES-32GF is presented in Table 1. It uses 361 slices and 1033 LUTs. The design does not use any block random access memory (BRAM) that is integral part of the FPGA. The S-box has been implemented in composite field to achieve better area utilization. As mentioned earlier, slice-M can be adapted to form the dynamic random-access memory (DRAMs) which can be used for the storage purposes. In our design, out of 361 slices that have been used; 42 percent are slice-M and 58 percent are slice-L. It has been depicted in Fig. 10. But slice-M are not used to form DRAM because of two reasons; first, s-box is implemented in GF(((2)^2^)^2^)^2^; second, key generation is on-the-fly; hence, no storage is required. Resource consumption have two major parts 292 slices are used for the purpose of Data-path implementation, which includes ShiftRows, SubBytes, MixColumns, Add Round key and feedback path, occupies 292 slices. Key Schedule occupies 52 slices. It has its own S-box for SubBytes calculation used in Key Expansion which consumes 22 slices. In total, design also consumes 395 slice registers and 110 flip flops.

The results obtained for the utilization of the resources are as follows:

The area savings are reported in Table 2, which presents the resource consumption of three designs. First is the AES-128, i.e. loop unrolled architecture for AES. It uses 128-bit data-path. Second is AES-32, the proposed design i.e. AES with 32 bit data path. Finally, AES with 32-bit input but 128-bit data path has also been implemented. All have been implemented on the same Artix-7 FPGA. Table 2 shows that AES-128 consumes 2668 slices and a total of 9571 LUTs. Hence, a total improvement of 86.47 percent is achieved by AES-32. Similarly, a comparison with AES-32 with 128-bit data path shows an improvement of 14.85 percent which is the extra cost of data path. This implementation consumes 424 slices and 1231 LUTs. Results of Table 2 are depicted in Fig. 8 which clearly emphasize the reduction achieved by the iterative design with compressed data path.

The design have been implemented on different FPGAs and corresponding results are compiled in the Table 3. Figs. 12 and 13 provides the graphic representation of the results of this table. Each figure present two parameters for a particular FPGA implementation. Fig. 12 presents the slice consumption and maximum frequency of operation and the throughput in Mbps for the design on these FPGAs. Here, we have used the complete name of the FPGA family like Spartan-3 or Virtex-5 etc. Fig. 13 presents and TPS in Mbps/slice for the design. The analysis of the results clearly show that Virtex-5 is the most suited FPGA for this design as it presents the most compact as well as most efficient implementation with the values of 595 slices and 3.368 Mbps/slice. The maximum frequency of operation and throughput is also achieved on this FPGA.

**Table 3:** A comparison of design performance on different FPGAs.

S. No.	Board	Slice	Frequency (MHz)	Throughput (Mbps)	TPS (Mbps/slice)
1.	Spartan-3	1271	58.361	747.0208	0.58774
2.	Virtex-4	1385	103.526	1325.1328	0.95677
3.	Virtex-5	595	156.594	2004.4032	3.36877498
4.	Spartan-6	583	87.298	1117.4144	1.9166
5.	Artix-7	765	140.292	1795.7376	2.3473

For the comparison with the existing designs, we have synthesized the design in Xilinx PlanAhead and implemented it on the different Xilinx FPGAs. It provided us with results to carry out a detailed comparison with existing designs. For comparison, we have adopted the strategy mentioned by [4] where the concept of ‘equivalent slices’ and a ‘normalization method’ was adopted to carry out the design comparison.

As the comparison is made among old and new devices, the following method has been utilized for the fair comparison. The concept of ‘equivalent slices’ is used for the same. As many designs are based on the look up tables which are stored in the BRAMs available in FPGA. Hence, the resource consumption has two parts: number of slices and BRAMs. The cost of a BRAM is calculated in term of slices and finally, added to number of slices to calculate the equivalent number of slices consumed by the design.

Detailed study of the literature suggests that different types of FPGA are used for implementation. FPGA used varies from Virtex-II to Virtex-7. Hence, a ‘normalized TPS’ calculation is provided for a fair comparison. This method is used from D.S. Kundi et al. Virtex-II, Virtex-II pro, Spartan-3, Virtex-E and Virtex-4 implementations are calculated under following assumptions:

(i) As a slice in these FPGAs comprises 2 LUTs while FPGAs from Virtex-5 onwards comprise of 4 LUTs per slice. Therefore, the occupied area is divided by 2 in case of these FPGAs.
(ii) In these FPGAs, one BRAM is equivalent to 64 slices (18 Kb BRAM) while Virtex-5 onwards the one BRAM is equivalent to 128 slices (36 Kb BRAM).
(iii) Finally, to normalize the frequency of operation, a factor of 1.22 (550/450) is multiplied to the frequencies achieved by these FPGAs because these FPGAs can have maximum frequency of operation 450 MHz while Virtex-5 onwards the maximum frequency of operation is 550 MHz.

Based upon this normalization criteria, the normalized frequency, throughput and TPS are calculated for the FPGAs of older generation i.e. before Virtex-5.

Table 4 presents the comparison of the proposed design with different compact AES implementations available in the literature. Again, the parameters of evaluation are same. The table presents the resource consumption in terms of equivalent slices which are calculated as per the criteria mentioned. Table 4 also use normalization procedure discussed to generate a fair comparison. It is necessary because it presents the designs from 2004 to 2020. Hence the FPGAs used are different. A total of 14 designs are compared here. Some utilizes BRAMs for the key storage and S-box implementations. It is very effective strategy in FPGA implementation as this relates to utilization of all FPGA resources. This strategy also generates faster output in comparison to the other design. Maximum frequency of operation also increases with the use of BRAMs. A particular design when implemented with BRAMs have clocked 1.12 to 3.5 times more frequency on the particular FPGA. So, it can be clearly stated that BRAM based design on FPGA operates at higher frequencies as compared to non-BRAM based designs. The acronyms used in the figures are the initials of the designs presented in Table 4 e.g. Zheng Yuan et al. (2011) have been written as ZY; Rourvoy et al. (2004) as R and Chodoweic and Gaj (2003) as CG etc. while the FPGAs are mentioned as V-4 or V-7 for Virtex-4 or 7 and S-2 or S-3 for spartan 2 or 3 respectively. These notations are chosen for the better management of space in Tables and figures.

**Table 4:** Comparison to various 32-bit implementations of AES.

Work	FPGA	Slice + BRAM	Equivalent Slices	Fmax (MHz)	Throughput (Mbps)	TPS (Mbps/Slice)
R [2]	S-3	163+3	355	71	208	0.70
CG [3]	S-2	222+3	414	60	166	0.32
DSK [4]	V-5	203+2	459	333	4262	9.29
RBH [5]	V-5	69+3	453	257	747	1.65
MEM [6]	V-5	303+10	1583	–	1330	0.425
SD [7](PBRAM) SBRAM	V-5	624+8 212+2	3296 936	550 550	6704 1676	2.034 1.784
GC [8]	V-6	415+16	4926	200	1815	0.368
LO [9]	V-E	3580	1790		157.07	0.08774
ZY [10]	V-5	885 4992	885 4992	103.3 116	300.4 1350	0.339 0.270
NB [11]	V-5	556	556	256	712.3	1.28
NM [12]	XCV800–4	4452	2226	28.06 (23)	29	0.013
NK [13]	V-4	2281	1141	167.14 (137)	–	–
UL [14]	S-3	287+3	479	123.464 (101.2)	294.4	0.61
HJ [15]	V-7	2444+0	2444	456	5306	2.17
TWV5	V-5	**595**	**595**	**156.594**	**2004.4032**	**3.37**
TWS3	S-3	**1271**	**636**	**58.361**	**747.0208**	**1.17548**

The results of Table 4 are depicted in Fig. 14, Fig. 15, Fig. 16. Fig. 14 shows the comparison of proposed design with other compact implementations on the basis of resource consumption. The most compact implementations are of [2] followed by [3]. Our design stands at seventh positions among these compact implementations. The resources have been minimized in comparison to eight other implementations. It shows that we are able to minimize the resource consumption compare to others designs but there is a scope for further reductions.

Fig. 15 shows the throughput comparison of the designs in Table 4. It is measured in Mbps. The detailed analysis reflects that parallel BRAM design by [7] generates the maximum throughput of 6704 Mbps followed by [15] and [4] at 5306 and 4926 respectively. Our design on V-5 stands at fourth position overall with 2004.40 Mbps.

On the other hand, with nearly 10 times more throughput generation, our implementation on Spartan-3 is way ahead from other two spartan implementations in throughput i.e., [2] and [3].

The real reflection of the effective design on FPGA is its efficiency. It is presented as throughput per slice (TPS) here and measured in Mbps/slice in the table. But the small values will not be differentiable in figure. Hence, Fig. 16 presents TPS of these designs in Kbps/slice for the better visibility and comparison. A quick glance on Fig. 16 shows that proposed design with 3370 Kbps/slice on Virtex-5 is second best after [4] which delivers a TPS of 9290 Kbps/slice. Though the design from [15] and [7] were ahead in throughput but in terms of efficiency this design outperforms these and other design with good margins. It signifies the optimum utilization of the resources by the proposed design.

Finally, we can say that after the design of [4], proposed design comes out second best in terms of performance among compact AES implementations.

**Fig. 9:**
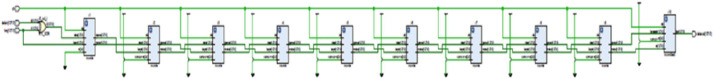
Top level Schematic for AES-128.

The results of Table 4 show the comparison of proposed design with other compact implementations on the basis of resource consumption. The most compact implementations are of [2] followed by [3]. Our design stands fifth among these compact implementations. The resources have been minimized in comparison to other implementations. It shows that these methods helped in minimizing the resource consumption compare to others designs but there is a scope for further reductions (Figs. 9 and 11).

**Fig. 10:**
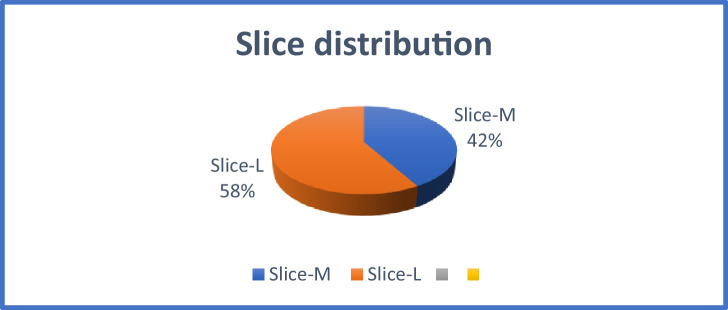
Slice distribution for AES-32.

**Fig. 11:**
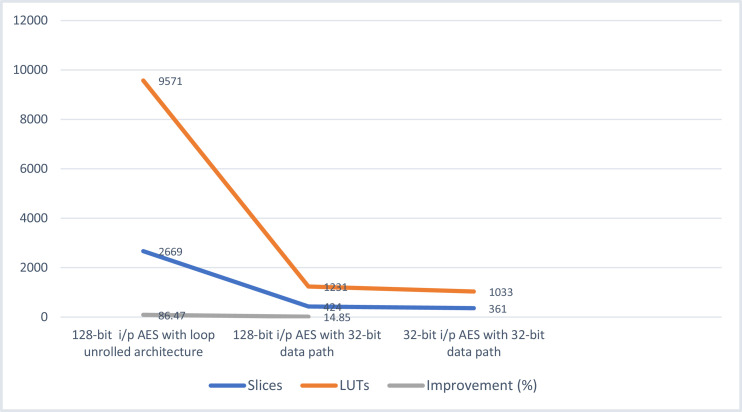
Comparison of three AES implementations from Table 3.

**Fig. 12:**
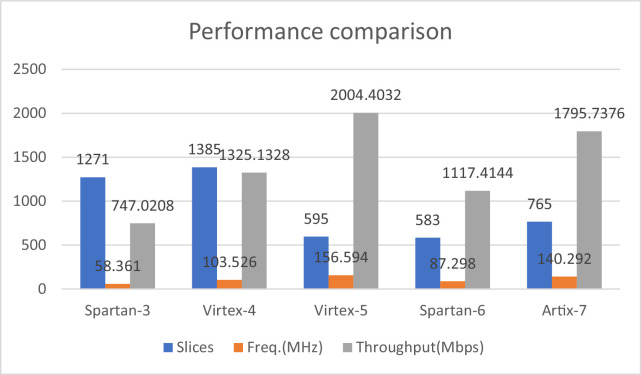
Area and Frequency performance comparison of AES-32GF on different FPGAs.

**Fig. 13:**
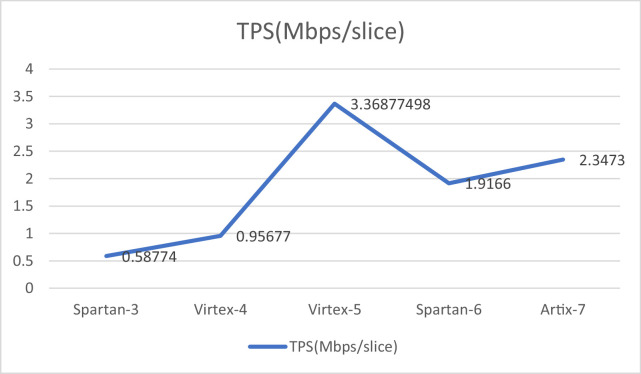
Throughput per slice comparison for AES-32GF on different FPGAs.

**Fig. 14:**
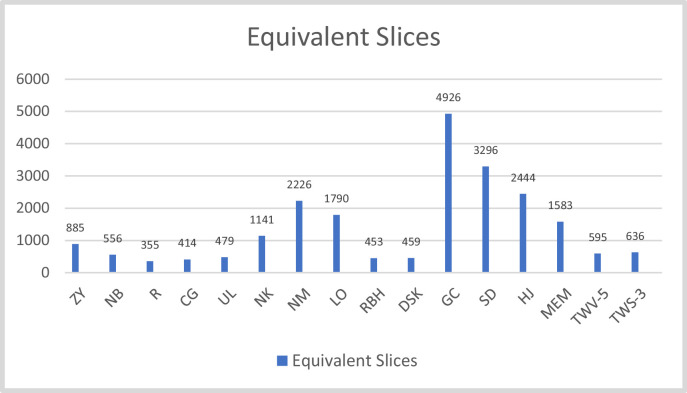
Comparison of the resource consumption with existing compact AES designs.

**Fig. 15:**
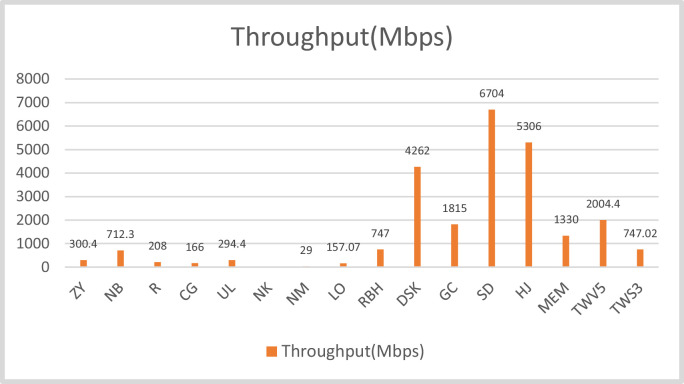
Throughput comparison of AES-32 with other compact AES designs.

**Fig. 16:**
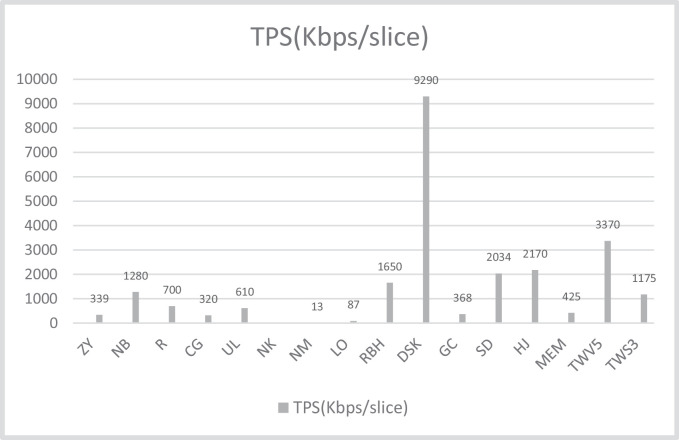
TPS comparison of AES-32 with other compact AES designs.

Throughput comparison of the designs in Table 4, measured in Mbps, shows that proposed design provides high throughput. The detailed analysis reflects that parallel BRAM design by [7] generates the maximum throughput of 6704 Mbps followed by [15] and [4] at 5306 and 4926 respectively. Our design on V-5 stands at fourth position overall with 2004.40 Mbps. The efficiency of the proposed design is high. It stands second with 3.37 Mbps/slice after [4].

## Benefits of AES-32 GF

Different applications in IoT call for different security solution. One type of solution can not fit in all the situations. As in case of vehicular or UAV communication, latency is critical parameter in design of cipher. Hence, a cipher must be designed for high throughput with low cycle count. In case of smart building application, the main concern is securing the small sensors and their information from hackers. Here, main parameter is to adapt cipher to these small devices. Hence, minimizing the resource consumption and area requirement should be the most important concern. The low area consumption (slice consumption) makes AES-32GF suitable for the implementation in the sensors which can be used for the information security in smart buildings in smart lighting, AC control etc. The most important feature of this implementation is that level of security has been maintained while minimizing the resource requirements. Its high throughput will help in case of surveillance applications.

## Limitations of AES-32 GF

AES-32GF has scope to reduce the resource-consumption further. It has latency of 10 cycles which makes it unsuitable for the applications such as real time vehicular control or smart-grid applications. With further reduction to size it can be made suitable to very small sensors as well.

## Ethics statements

We have adhered to MethodsX ethical guidelines.

## CRediT authorship contribution statement

**Sumit Singh Dhanda:** Conceptualization, Methodology, Writing – original draft, Software. **Poonam Jindal:** Visualization, Investigation, Writing – review & editing. **Brahmjit Singh:** Supervision, Writing – review & editing. **Deepak Panwar:** Writing – original draft, Software.

## Declaration of Competing Interest

The authors declare that they have no known competing financial interests or personal relationships that could have appeared to influence the work reported in this paper.

## Data Availability

Data will be made available on request.
